# Triagem de Deficiência de Lipase Ácida Lisossômica em uma Unidade Clínica de Dislipidemias

**DOI:** 10.36660/abc.20240825

**Published:** 2025-07-29

**Authors:** Zenia Brasil, Francisco Antonio H. Fonseca, Marco Antonio Curiati, Sandra Obikawa Kyosen, Vanessa Gonçalves Pereira, Waleria Toledo Fonzar, João Bosco Pesquero, Francy Reis da Silva Patrício, Marcelo Hideki Yamamoto, Joyce Umbelino Yamamoto, Vania D’Almeida, Ana Maria Martins, Maria Cristina Izar

**Affiliations:** 1 Departamento de Medicina Universidade Federal de São Paulo São Paulo SP Brasil Disciplina de Cardiologia, Departamento de Medicina, Universidade Federal de São Paulo (UNIFESP), São Paulo, SP – Brasil; 2 Hypertri Brazil Network Casa dos Raros Porto Alegre, RS Brasil Brazilian Network of Collaboration and Knowledge Advancement on Severe Hypertriglyceridemia – Hypertri Brazil Network, Casa dos Raros, Porto Alegre, RS – Brasil; 3 Centro de Referência em Erros Inatos do Metabolismo Departamento de Pediatria Universidade Federal de São Paulo São Paulo SP Brasil Centro de Referência em Erros Inatos do Metabolismo (CREIM), Departamento de Pediatria, Universidade Federal de São Paulo (UNIFESP), São Paulo, SP – Brasil; 4 Laboratório de Erros Inatos do Metabolismo Universidade Federal de São Paulo São Paulo SP Brasil Laboratório de Erros Inatos do Metabolismo, Universidade Federal de São Paulo (UNIFESP), São Paulo, SP – Brasil; 5 Departamento de Biofísica Universidade Federal de São Paulo São Paulo SP Brasil Departamento de Biofísica, Universidade Federal de São Paulo, São Paulo, SP – Brasil; 6 Departamento de Patologia Universidade Federal de São Paulo São Paulo SP Brasil Departamento de Patologia, Universidade Federal de São Paulo (UNIFESP), São Paulo, SP – Brasil

**Keywords:** Lipase, Dislipidemias, Hepatomegalia, Esplenomegalia

## Abstract

**Fundamento:**

A deficiência da lipase ácida lisossômica (D-LAL) é uma doença autossômica recessiva rara, caracterizada pelo acúmulo maciço de ésteres de colesterol e triglicerídeos em vários órgãos, levando à hepatosplenomegalia, esteatose microvesicular, cirrose e morte prematura. O reconhecimento precoce é crucial para a realização da terapia de reposição enzimática em tempo hábil.

**Objetivos:**

Rastrear a D-LAL em indivíduos com dislipidemias e/ou doença hepática em uma unidade clínica de dislipidemias.

**Métodos:**

Avaliamos retrospectivamente registros de 2018 adultos e crianças utilizando um algoritmo de rastreamento que incluía elevação de ALT/AST >1,5x o limite superior da normalidade, LDL-C >160 mg/dL, HDL-C <40 mg/dL (homens) ou <50 mg/dL (mulheres) em adultos, e LDL-C >130 mg/dL, HDL-C <45 mg/dL em crianças. Pacientes de alto risco para D-LAL foram selecionados para ensaio de atividade enzimática da LAL em amostras de sangue seco, utilizando o inibidor de LAL, Lalistat-2.

**Resultados:**

Entre 2018 pacientes rastreados, 21 (0,92%) foram selecionados para avaliação da atividade de LAL, mas apenas oito apresentaram resultados normais no teste [atividade média de LAL 0,077 ± 0,03 nmol/punção/h (valor de referência >0,024 nmol/punção/h)]. Uma criança cuja mãe não realizou o teste teve atividade de LAL indetectável em exame *post-mortem.* Posteriormente, a mãe e três meio-irmãos tiveram a confirmação de D-LAL. O sequenciamento genético (NGS) do gene LIPA não identificou variantes patogênicas, o que não permite descartar alterações na região não codificante do gene analisado.

**Conclusões:**

A identificação da D-LAL continua sendo um desafio, e um algoritmo baseado em critérios clínicos e laboratoriais pode auxiliar na seleção de pacientes para rastreamento da D-LAL. Dada a sua raridade e características comuns a outras dislipidemias genéticas, a D-LAL é, principalmente, um diagnóstico de exclusão, frequentemente considerado quando outras condições tenham sido descartadas.

## Graphical abstract

**Figura Central:**
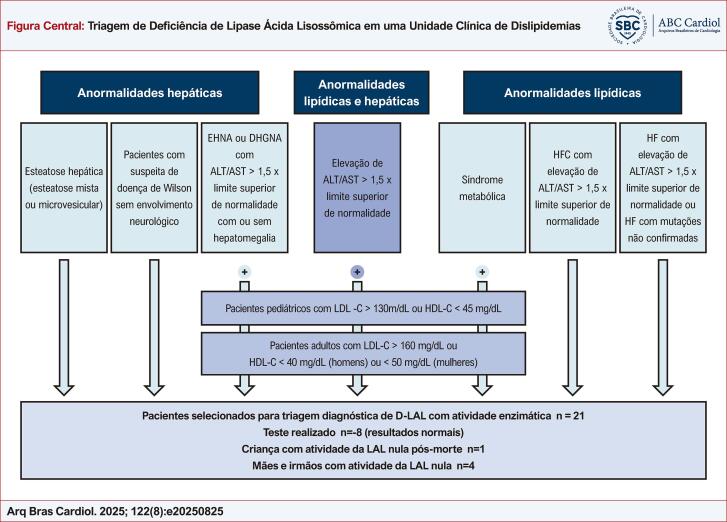
Triagem de Deficiência de Lipase Ácida Lisossômica em uma Unidade Clínica de Dislipidemias Algoritmo proposto para triagem de D-LAL utilizando atividade enzimática, de acordo com anormalidades hepáticas e/ou lipídicas, adicionando a trajetória do paciente até o diagnóstico. Critérios de inclusão: i) Adulto, não obeso (IMC <30 kg/m^2^), com LDL-C >160 mg/dL, colesterol de lipoproteína de alta densidade (HDL-C) <40 mg/dL em homens e <50 mg/dL em mulheres, e com níveis persistentemente elevados de Alanina Aminotransferase (ALT) e/ou Aspartato Aminotransferase (AST), >1,5 vezes o limite superior da normalidade, com ou sem hepatomegalia; ii) Pacientes com esteatose detectada por ultrassonografia ou esteatose mista ou microvesicular observada em biópsia hepática; iii) Pacientes com suspeita de HF utilizando os critérios da Dutch Lipid Clinic Network, sem confirmação de variantes causadoras de HF no sequenciamento, ou com variantes no gene LIPA, com ALT e/ou AST >1,5 vezes o limite superior da normalidade; iv) pacientes pediátricos (<18 anos) com hepatomegalia detectada por exame físico ou exames de imagem (com ou sem esplenomegalia), LDL-C elevado (>130 mg/dL), HDL-C baixo (<40 mg/dL), ou níveis persistentemente elevados de ALT e/ou AST (>1,5x o limite superior da normalidade), e/ou sinais de fibrose, cirrose ou evidência de doença de armazenamento em biópsia hepática; v) Pacientes com suspeita de Doença de Wilson sem envolvimento neurológico; HFC: hiperlipidemia familiar combinada; HF: hipercolesterolemia familiar; EHNA: esteatose hepática não alcoólica; DHGNA: doença hepática gordurosa não alcoólica.

## Introdução

A lipase ácida lisossômica (LAL) é a enzima responsável pela hidrólise intracelular de ésteres de colesterol e triglicerídeos.^1^ A deficiência da LAL (D-LAL) é caracterizada pelo acúmulo de ésteres de colesterol e triglicerídeos, mas também leva ao aumento da síntese de colesterol.^2^ A D-LAL (OMIM #278000) é uma doença rara de armazenamento lisossômico autossômica recessiva, decorrente de variantes no gene LIPA (OMIM 613497), mapeado no cromossomo 10q23.2, com 10 éxons e aproximadamente 45 kb de comprimento.^3^ Os indivíduos afetados são normalmente homozigotos ou heterozigotos compostos para variantes do gene LIPA.

A D-LAL manifesta-se em duas formas distintas. A doença de Wolman,^4^ em lactentes, apresenta sintomas e sinais agressivos, como acúmulo maciço de ésteres de colesterol e triglicerídeos no fígado, baço, medula óssea, glândulas suprarrenais, linfonodos, vilosidades intestinais, além de infiltração no endotélio vascular e no músculo esquelético. A calcificação nas glândulas suprarrenais é uma característica comum da LAL-D e pode ocorrer em 50% dos lactentes.^5 , 6^ Falha no crescimento decorrente de má absorção, hepatomegalia e esplenomegalia, além da insuficiência hepática devido a fibrose e cirrose, podem estar presentes. Sem tratamento, essas complicações contribuem para o óbito no primeiro ano de vida.^7 , 8^ Estudos de prevalência que avaliam esse fenótipo grave são escassos e variam de acordo com etnia e localização geográfica, indo de 1:350.000 a 1:528.000 em crianças, com atividade LAL ausente ou inferior a 1%, sendo geralmente indivíduos homozigotos para variantes patogênicas do gene LIPA.^9^

Outro fenótipo da LAL-D é a Doença de Depósito de Ésteres de Colesterol (CESD, do inglês *cholesteryl ester storage disease* ),^10^ que apresenta uma variação na manifestação clínica, dependendo da atividade da LAL, variando de 1 a 12% do valor normal.^11^ Esse subtipo é menos reconhecido, com progressão lenta, e pode ser confundido com outras doenças que apresentam achados clínicos e laboratoriais semelhantes, como hipercolesterolemia familiar (HF), hiperlipidemia familiar combinada (HFC), Esteato-Hepatite Associada à Disfunção Metabólica (MASH, do inglês *metabolic dysfunction-associated steatohepatitis* ), doença hepática gordurosa associada ao metabolismo (DHGAM) ou cirrose criptogênica.^12^ Esse fenótipo geralmente ocorre devido a variantes patogênicas homozigóticas ou heterozigóticas compostas, ou pode ser atribuído a um alto escore poligênico; os indivíduos afetados não apresentam sinais de CESD até a infância ou idade adulta.^13^ As principais características da CESD incluem distensão abdominal, varizes esofágicas, hepatosplenomegalia, doença arterial coronariana, diarreia, acidente vascular cerebral, desnutrição e falha no crescimento.^5^ Bernstein et al.^6^ descobriram que a disfunção hepática e a dislipidemia foram os sinais mais frequentes em todas as idades na CESD, sendo a hepatomegalia e a hepatosplenomegalia os sinais mais comuns da doença.

Em relação ao tratamento, a terapia de redução de lipídios com estatinas parece ineficaz para pacientes com distúrbios hepáticos, pois aumenta o fornecimento de ésteres de colesterol para os hepatócitos,^13 , 14^ agravando, assim, a doença.^15^ No entanto, trata-se d melhor opção para reduzir as concentrações de colesterol de Lipoproteína de Baixa Densidade (LDL-C), a síntese de colesterol e o risco cardiovascular.^16^ Além disso, na LAL-D, há uma regulação deficiente do gene ABCA1, levando à redução nas concentrações de HDL-C.^17^ A terapia de reposição enzimática com sebelipase alfa permite que os pacientes atinjam níveis fisiológicos de LAL e pode prevenir o acúmulo de ésteres de colesterol e triglicerídeos.^4 , 18^

Alguns países desenvolveram diretrizes para aumentar a conscientização e o diagnóstico da D-LAL.^19^ Em nosso país,^19^ a D-LAL foi incluída como uma doença lisossômica, e nós seguimos um algoritmo de diagnóstico ( Figura Central ), previamente utilizado como ferramenta de rastreio.^20^ Neste artigo, descrevemos os métodos de rastreamento para detectar D-LAL em crianças e adultos em uma clínica terciária para tratamento de dislipidemias. Buscamos sinais de doença hepática, disfunção hepática associada à dislipidemia ou alterações lipídicas isoladas. Esse método de rastreamento foi baseado em diagnósticos anteriores de pacientes com D-LAL confirmada, selecionados a partir de um caso índice pós-morte com familiares afetados no Centro de Referência em Erros Inatos do Metabolismo (CREIM), da Universidade Federal de São Paulo.^20^

O objetivo deste estudo foi investigar a prevalência de D-LAL em uma população de pacientes com dislipidemias, disfunção hepática, ou ambos, em tratamento ambulatorial, com base em casos prévios confirmados pelo mesmo método de rastreamento.

## Métodos

### Delineamento do estudo

Este é um estudo retrospectivo transversal, com dados obtidos a partir de registros médicos (em papel e/ou eletrônicos) de pacientes adultos e pediátricos das unidades clínicas de dislipidemias, Aterosclerose e Biologia Vascular, Divisão de Cardiologia, da Universidade Federal de São Paulo. O protocolo do estudo foi aprovado pelo nosso comitê de ética local (Comitê de Ética em Pesquisa da UNIFESP, CAAE: 51989915.2.00005505), e os dados foram coletados ao longo de 18 meses.

Revisamos 2000 registros médicos consecutivos de pacientes adultos e 18 pacientes pediátricos entre agosto de 2017 e fevereiro de 2019. Entre eles, havia 92 pacientes com diagnóstico definitivo/provável de HF (Dutch Lipid Clinic Network), mas com resultados negativos para um painel de genes relacionados à HF (LDLR, APOB, PCSK9, LDLRAP-1), além do gene LIPA,^21^ e 168 pacientes com doença hepática. Dados clínicos e laboratoriais desses pacientes foram obtidos para rastreamento seletivo de D-LAL.

Critérios de inclusão

- Adultos, não obesos (Índice de Massa Corporal, IMC <30 Kg/m^2^), com LDL-C >160 mg/dL, lipoproteína de alta densidade (HDL-C) <40 mg/dL em homens e <50 mg/dL em mulheres, e com níveis de alanina aminotransferase (ALT) e/ou aspartato aminotransferase (AST) persistentemente elevados (>1,5 vezes o limite superior da normalidade), com ou sem hepatomegalia.
- Pacientes com esteatose detectada por ultrassonografia ou esteatose mista ou microvesicular observada em biópsia hepática.
- Pacientes com suspeita de HF usando os critérios da Dutch Lipid Clinic Network,^22^ sem confirmação de variantes causadoras de HF na análise genética, ou com variantes no gene LIPA, e níveis de ALT e/ou AST >1,5 vezes o limite superior da normalidade.
- Pacientes pediátricos (<18 anos) com hepatomegalia detectada no exame físico ou por exames de imagem (com ou sem esplenomegalia), LDL-C elevado (>130 mg/dL), HDL-C baixo (<40 mg/dL), ou níveis persistentemente elevados de ALT e/ou AST (>1,5 vezes o limite superior da normalidade), e/ou sinais de fibrose, cirrose ou evidência de doença de armazenamento em biópsia hepática.
- Pacientes com suspeita de doença de Wilson sem envolvimento neurológico.

Critérios de exclusão

- Pacientes adultos com esteatose ou cirrose de etiologia conhecida;
- Pacientes com hepatite viral e/ou doença hepática alcoólica;
- Pacientes com toxicidade conhecida por medicamentos.

### Organização do banco de dados

Os dados coletados incluíram identificação dos pacientes, dados demográficos, presença de fatores de risco cardiovascular, incluindo dislipidemia, hábitos, história de doença cardiovascular, doenças hepáticas, síndrome metabólica e outras comorbidades. Foram registrados parâmetros clínicos e antropométricos. As variáveis laboratoriais incluíram colesterol total, HDL-C, LDL-C, não-HDL-C, Triglicerídeos (TG), ALT, AST, glicemia, Hormônio Estimulante da Tireoide (TSH) e Tiroxina Livre (T4L). Nos indivíduos em uso de estatinas, o LDL-C foi corrigido conforme a intensidade da estatina, conforme descrito abaixo.^23^ Ultrassonografias de fígado e baço, assim como biópsias hepáticas, foram registradas para identificação de anormalidades, quando disponíveis. Testes laboratoriais prévios ou confirmação genética de LAL-D também foram registrados, quando disponíveis. Para pacientes pediátricos, foi criado um banco de dados separado.

### Tratamento com estatinas

Os valores de colesterol total e LDL-C dos pacientes adultos foram corrigidos de acordo com a intensidade do tratamento com estatinas, multiplicando-se os valores de colesterol total e LDL-C por um fator: alta intensidade, redução do LDL-C >50% (x 2,00); intensidade moderada, redução do LDL-C entre 30-50% (x 1,65); e baixa intensidade, redução do LDL-C ~30% (x1,43).^23^O tratamento de alta intensidade foi definido como atorvastatina 40/80 mg, rosuvastatina 20/40 mg, sinvastatina 20/40 mg mais ezetimiba. O tratamento de intensidade moderada como atorvastatina 10/20 mg, rosuvastatina 5/10 mg, sinvastatina 10/40 mg, e o tratamento de baixa intensidade definido como outras estatinas ou doses menores.

### Diagnóstico bioquímico

**Figura 1:**
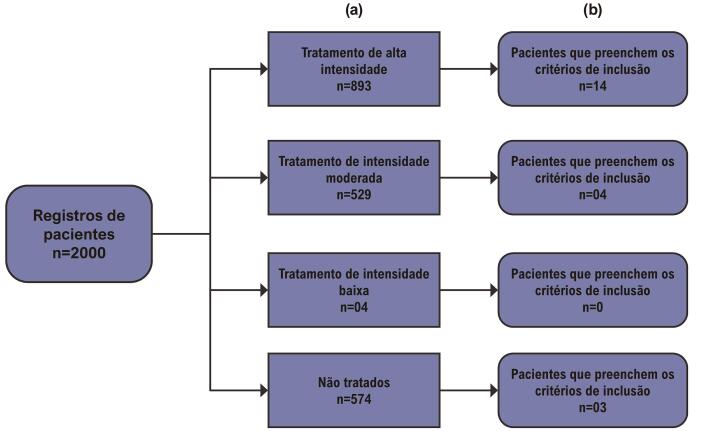
– Perfil de pacientes com dislipidemia a partir de prontuários médicos, de acordo com a intensidade do tratamento com estatinas (a) e aqueles com critérios de inclusão para LAL-D (b). Alta intensidade: atorvastatina 40/80 mg, rosuvastatina 20/40 mg, sinvastatina 20/40 mg + ezetimiba 10 mg; intensidade moderada: atorvastatina 10/20 mg, rosuvastatina 5/10 mg, sinvastatina 10/40 mg; baixa intensidade: outras estatinas ou doses menores.

Os indivíduos que preencheram os critérios de inclusão/exclusão foram convidados a coletar amostras de sangue para determinação da atividade da LAL ( Figura 1 ). Esses pacientes assinaram o termo de consentimento informado por escrito antes da coleta de sangue.

O ensaio de atividade enzimática da LAL foi realizado a partir de amostras de sangue seco, seguindo-se a técnica fluorimétrica descrita por Hamilton et al.,^24^ no Laboratório de Erros Inatos do Metabolismo (LEIM) – UNIFESP. Foi utilizado o intervalo de referência de >0,024 nmol/punção/h. O ensaio foi realizado em duplicata, com controles positivos e negativos.

### Estudo genético para detecção de hipercolesterolemia familiar

A extração do DNA, o sequenciamento de nova geração (NGS, do inglês *next generation sequencing* ) e as análises de detecção de variantes foram realizados de acordo com Fonzar et al.^21^

### Análise dos dados

Neste estudo, utilizou-se a estatística descritiva. As variáveis categóricas foram descritas como frequências e percentuais; os dados numéricos foram apresentados como médias ± desvio padrão (DP) ou mediana e intervalo interquartil (IQR), conforme apropriado. A normalidade foi testada pelo teste de Kolmogorov-Smirnov. A significância foi estabelecida com um valor de p < 0,05. Todas as análises estatísticas foram realizadas utilizando o *Statistical Package for the Social Sciences* (SPSS, Chicago, IL), versão 22.

## Resultados

### Análise descritiva da população adulta

As características da população adulta estão apresentadas na Tabela 1 e incluíram a coorte de adultos com HF. Dos 2000 registros de pacientes avaliados, 893 indivíduos (44,6%) estavam recebendo tratamento de alta intensidade, 529 (26,4%) tratamento de intensidade moderada, quatro (0,2%) tratamento de baixa intensidade, e 574 (28,8%) não estavam tomando estatinas. Desses, 21 pacientes atenderam aos critérios de inclusão para a avaliação da atividade de LAL ( Figura 1 ), sendo 14 em tratamento de alta intensidade, quatro em tratamento de intensidade moderada e três sem uso de estatinas.

**Tabela 1:** – Características clínicas e laboratoriais dos pacientes adultos

Características	N=2000
Idade, anos	67 (60-74)
Sexo masculino/feminino, %	613 (30,65) / 1387 (69,35)
Raça, %	
Caucasiana	1022 (51,10)
Preta	197 (9,85)
Mista	781 (39,05)
Hipertensão, %	1528 (76,4)
Dislipidemia, %	1615 (80,75)
Diabetes, %	778 (38,9)
Síndrome metabólica, %	595 (29,75)
Intensidade da estatina, %	
Alta intensidade	893 (44,6)
Intensidade moderada	529 (26,4)
Baixa intensidade	4 (0,2)
Sem estatina	574 (28,8)
Infarto do miocárdio	215 (10,75)
Tabagismo, %	
Atual	110 (5,50)
Prévio	467 (23,25)
IMC, kg/m^2^	30,88 (24,65-40,00)
Colesterol total, mg/dL	227 (152-279)
HDL-C, mg/dL	49 (40-59)
LDL-C, mg/dL	156 (115-204)
Não HDL-c, mg/dL	121 (96-155)
Triglicerídeos, mg/dL	124 (99-177)
AST, U/L	48 (48-60)
ALT, U/L	49 (49-61)
Glicose, mg/dL	102 (94-118)
HbA1c, %	6,10 (5,70-6,70)
TSH, mUI/L	2,65 (1,76-4,21)
T4, ng/dL	1,30 (1,14-1,51)

As variáveis categóricas são expressas como frequências (%); as variáveis numéricas são expressas como mediana (IQR); a correção para a intensidade do tratamento foi feita multiplicando o colesterol total ou LDL-C por 1,43 (baixa intensidade), 1,65 (intensidade moderada) e 2,00 (alta intensidade); HDL-C: lipoproteína de alta densidade; LDL-C: lipoproteína de baixa densidade; ALT: alanina aminotransferase; AST: aspartato aminotransferase; IQR: intervalo interquartil; TSH: hormônio estimulante da tireoide; T4L: tiroxina livre; IMC: índice de massa corporal.

A coorte de 92 pacientes com diagnóstico definitivo ou provável de HF (Dutch Lipid Clinic Network) também foi selecionada.^22^ Entre esses, encontramos dois pacientes com variantes de significado incerto no gene LIPA, enzimas hepáticas normais e sem sinais de esteatose hepática. Esses pacientes não foram rastreados quanto a atividade de LAL.

### Análise descritiva dos pacientes pediátricos com critérios para D-LAL

Dezoito pacientes foram identificados nos prontuários médicos da nossa unidade clínica de dislipidemias e do CREIM ( Tabela 2 ). Desses, oito (44%) tinham LDL-C acima do alvo, e três (17%) apresentavam HDL-C <40 mg/dL; nove pacientes apresentaram níveis elevados de AST e/ou ALT. Todos os critérios estavam presentes em cinco (28%) pacientes. Um desses pacientes teve confirmação genética para a doença de Niemann-Pick, outro possuía uma doença mitocondrial hereditária e não foram selecionados para o rastreamento de D-LAL.

**Tabela 2:** – Características das crianças rastreadas para deficiência de lipase ácida lisossômica

Variável	Total (N=18)
Idade, anos	12 (7-18)
Masculino/feminino, %	12 (67)/6 (33)
Raça, %	
Caucasiana	10 (55)
Preta	1 (6)
Mista	7 (39)
Terapia hipoglicemiante, %	
Alta intensidade	0 (0)
Intensidade moderada	1 (5)
Baixa intensidade / nenhuma terapia	1 (5)
Sem estatina	16 (90)
Peso corporal, kg	38 (26-48)
IMC, Kg/m^2^	18 (17-19)
Frequência cardíaca, bpm	70 (67-76)
Colesterol total, mg/dL	213 (135-329)
HDL-C, mg/dL	44 (21-64)
LDL-C, mg/dL	152 (67-250)
Não HDL-C, mg/dL	170 (82-265)
Triglicerídeos, mg/dL	91 (52-167)
Glicemia de jejum, mg/dL	88 (77-103)
HbA1c, %	5,5 (4,8-6,3)
AST (U/L)	27 (18-66)
ALT (U/L)	19 (10-62)
CK (U/L)	135 (110-241)
TSH (µIU/L)	3,30 (1,24-6,12)

As variáveis categóricas são expressas como frequências (%); as variáveis numéricas são expressas como mediana (IQR); ALT: alanina aminotransferase; AST: aspartato aminotransferase; IMC: índice de massa corporal; HDL: lipoproteína de alta densidade; IQR: intervalo interquartil; LDL: lipoproteína de baixa densidade; DP: desvio padrão; TSH: hormônio estimulante da tireoide; CK: creatinofosfoquinase.

### Análise descritiva dos pacientes com esteatose hepática

A Tabela 3 apresenta as características de 168 pacientes com esteatose hepática, detectada por ultrassonografia ou biópsia hepática. Cento e cinquenta e cinco (92,26%) pacientes apresentaram esteatose na ultrassonografia, hepatopatia parenquimatosa foi encontrada em 12 (7,14%), e nódulo hepático em 1 (0,59%). A partir desses achados, selecionamos pacientes com LDL-C corrigido >160 mg/dL e HDL-C <40 mg/dL em homens ou <50 mg/dL em mulheres, AST ou ALT >1,5x o limite superior da normalidade ou esteatose hepática detectada por métodos de imagem.

**Tabela 3:** – Distribuição dos achados da ultrassonografia e/ou da biópsia hepática nos participantes do estudo

Variável	N=168
Esteatose (%)	155 (92,26)
Hepatopatia parenquimatosa (%)	12 (7,14)
Nódulo hepático (%)	1 (0,59)

Variáveis categóricas expressas em frequências (%).

A análise de pacientes com doença de Wilson sem envolvimento neurológico não foi realizada, pois não havia indivíduos com esse critério.

### Atividade da LAL

Dos bancos de dados selecionados, 21 (0,92%) pacientes atenderam aos critérios de inclusão para o rastreamento de D-LAL com atividade de LAL (três crianças e 18 adultos); as médias de ALT/AST foram, respectivamente, 83,7 e 96,7 U/L; a média do colesterol total foi 248 mg/dL, do LDL-C 222 mg/dL, e quatro apresentaram esteatose hepática documentada. Desses, dois indivíduos não concordaram em participar, três foram a óbito, e não foi possível contatar oito pacientes, incluindo as crianças. Oito pacientes assinaram o termo de consentimento informado e coletaram sangue para o ensaio. Seis eram mulheres e dois homens com idades entre 58-82 anos; em 100% dos casos, os níveis de ALT estavam acima de 1,5 vezes o limite superior da normalidade, dois apresentavam esteatose hepática e todos estavam em tratamento com estatinas de alta intensidade. Não foram encontradas diferenças significativas entre os pacientes selecionados e triados para LAL-D (Tabela Suplementar S1).

O ensaio da atividade de LAL foi realizado em duplicata, além de dois controles positivos e dois controles negativos. A atividade média de LAL foi normal [0,077 (± 0,03) nmol/punção/h, valor de referência >0,024 nmol/punção/h]. Os controles negativos apresentaram 0,096 nmol/punção/h e os controles positivos mostraram atividade de LAL indetectável ( Tabela 4 ).

**Tabela 4:** – Resultados da atividade da lipase ácida lisossômica no teste enzimático utilizando o inibidor Lalistat-2

Amostra	Poço	Valores	Média	DP	V%	Sem - Com	pmol/punção/L	Linha reta 4/11
01	C2 D2	2698,469 2714,956	2706,713	11,658	0,4	332,211	40,991	45,558
02	C3 D3	2794,832 2643,668	2719,250	106,889	3,9	488,673	62,988	73,908
03	C4 D4	2835,840 2881,884	2858,862	32,558	1,1	702,193	92,940	112,511
04	C5 D5	2867,881 2931,860	2899,871	45,240	1,6	651,496	85,885	103,418
05	C6 D6	2707,930 2725,490	2716,710	12,417	0,5	546,769	71,117	84,385
06	C7 D7	2781,802 2832,713	2807,258	36,000	1,3	602,909	78,958	94,490
07	C8 D8	2782,242 2791,745	2786,994	6,720	0,2	608,951	79,848	95,638
08	C9 D9	2824,358 2847,077	2835,718	16,065	0,6	776,723	103,405	125,998
+ Controle	G10 H10	2693,484 2824,164	2758,824	92,405	3,3	-40,715	-11,371	-21,928
- Controle	G9 H9	2806,659 2734,641	2770,650	50,924	1,8	729,464	96,769	117,446

Os testes foram realizados em duplicata para cada paciente, incluindo um controle positivo (+ Controle) e um controle negativo (- Controle); a atividade de lipase ácida lisossômica foi avaliada com o inibidor Lalistat-2, e os resultados foram expressos em nmol/punção/h (valor de referência >0,024 nmol/punção/h).

Tentamos obter mais detalhes sobre esses pacientes, e uma mulher de 41 anos, cuja atividade de LAL era previamente normal, repetiu o teste de atividade de LAL após a morte de um de seus filhos. A atividade de LAL era 0,00 nmol/punção/h em dois testes posteriores. A paciente apresentou hepatomegalia e esteatose hepática, confirmando o diagnóstico de D-LAL. Sua filha de nove anos tinha hepatosplenomegalia e esteatose hepática, com níveis de AST de 55 e ALT 35 U/L, colesterol total e LDL-C de 212 mg/dL e 148 mg/dL, respectivamente; HDL-C de 12 mg/dL, não-HDL-C de 200 mg/dL e triglicerídeos de 256 mg/dL. Os sintomas começaram aos três anos de idade, com episódios intermitentes de diarreia e falha no crescimento. Aos quatro anos, seu peso corporal era de 16 kg (z score de −0,56) e sua altura era de 93 cm (z score de −3,0). A biópsia hepática mostrou esteatose difusa macro e microvesicular de grau 3 em 60% dos hepatócitos, aumento dos espaços portais com macrófagos microvacuolizados aumentados e hepatócitos. Aos nove anos, ela desenvolveu hepatosplenomegalia progressiva e piora dos episódios de diarreia. Foi hospitalizada várias vezes devido a insuficiência respiratória secundária à hepatosplenomegalia grave e faleceu aos nove anos devido a pneumonia e choque séptico.

O diagnóstico diferencial de D-LAL da criança incluiu a análise de amostras de sangue e biópsias hepáticas. Três biópsias hepáticas realizadas em diferentes hospitais mostraram esteatose macro e microvesicular, com um padrão semelhante ao da doença de Niemann-Pick tipo C.

Amostras de sangue foram coletadas, mas analisadas post-mortem. Os testes foram desenvolvidos para avaliar a atividade enzimática associada a diversas doenças de armazenamento lisossômico. A atividade da quitotriosidase foi medida no plasma utilizando um ensaio imunoenzimático (ELISA); a atividade da esfingomielinase foi avaliada em leucócitos para excluir a doença de Niemann-Pick; a atividade da beta-glicosidase foi medida em leucócitos para avaliar a doença de Gaucher; um painel abrangente obtido a partir de amostras de sangue seco testou quitotriosidase, esfingomielinase, beta-glicosidase e LAL, fornecendo uma avaliação mais ampla para doenças de armazenamento lisossômico. Todos os testes, exceto para D-LAL, estavam dentro da faixa de normalidade. Foi observada atividade enzimática indetectável (0 nmol/punção/h) (faixa de referência >0,024 nmol/punção/h), confirmando o diagnóstico de deficiência de LAL. Esses ensaios enzimáticos foram essenciais para diferenciar LAL-D de outras doenças de armazenamento lisossômico com características clínicas sobrepostas.

A autópsia revelou acúmulo disseminado de lipídios e cristais de colesterol no fígado, baço, medula óssea e trato gastrointestinal, além de esteatose grave com histiócitos preenchidos por lipídios. Não foi observada calcificação adrenal. Este foi o caso índice de sua família, sendo a segunda de cinco irmãos da mesma mãe, que teve um filho (não afetado) e uma filha (essa paciente) de seu primeiro casamento, e três outros filhos (todos afetados) de seu segundo casamento. Ambos os casamentos foram uniões não consanguíneas, e os pais não possuem parentesco entre si.

Os três meio-irmãos do sexo masculino foram avaliados, e eles apresentaram atividade de LAL de 0,0087 nmol/punção/h, 0,00 nmol/punção/h e 0,00 nmol/punção/h, confirmando o diagnóstico de D-LAL. Seus relatos de caso foram publicados anteriormente.^20^Além disso, realizamos testes genéticos da mãe e dos três meio-irmãos do caso índice, obtendo DNA extraído da saliva. O sequenciamento genético (NGS), utilizando um painel que incluía o gene LIPA, não encontrou variantes patogênicas, o que não permitiu descartar alterações na região não codificante dos genes analisados, como regiões regulatórias, sequências intergênicas e intrônicas distantes dos éxons.

## Discussão

Neste estudo, investigamos a prevalência de D-LAL em uma população de pacientes com dislipidemias em seguimento ambulatorial, e utilizamos um algoritmo baseado em casos clínicos previamente confirmados, diagnosticados por meio do ensaio enzimático para atividade da LAL.

A doença é considerada muito rara, com acúmulo de ésteres de colesterol em tecidos como fígado, baço, medula óssea, glândulas suprarrenais, linfonodos, vilosidades intestinais, parede vascular e músculos esqueléticos.^5 , 6^ Uma forma mais grave é reconhecida em crianças, e manifestações menos pronunciadas podem ser observadas em adultos, dependendo da atividade enzimática da LAL.^4^Em nosso estudo, na clínica de lípides, entre 2.018 indivíduos, identificamos 21 (0,92%) com alto risco para LAL-D. Dos oito inicialmente testados para atividade da LAL, nenhum caso foi confirmado. No entanto, um teste post-mortem positivo em uma criança (caso índice) permitiu confirmar a mãe e três meio-irmãos afetados.

A Atualização das Diretrizes Brasileiras para Dislipidemia e Prevenção da Aterosclerose^19^ reconhece a lacuna no diagnóstico da doença e sugere um algoritmo para triagem da D-LAL ( Figura Central ). Indivíduos com D-LAL enfrentam uma grande carga da doença e, segundo seus relatos, os sintomas são constantes, incluindo dor abdominal, hepatosplenomegalia, cefaleia, fraqueza, prurido e lesões cutâneas.^20^ Em crianças, o fenótipo pode estar presente nos primeiros meses de vida, com aumento abdominal, falha no crescimento, hepatomegalia ou hepatosplenomegalia, esteatose hepática e calcificação adrenal.^20 , 25^

Existe um amplo espectro de manifestações da doença de início tardio. Os sintomas podem ser leves e não percebidos, levando ao subdiagnóstico. Por outro lado, nos casos mais graves, as alterações hepáticas podem resultar em cirrose hepática e morte.^5^ Além disso, pode existir um risco cardiovascular aumentado, com histórico de doença cardiovascular aterosclerótica ou acidente vascular cerebral isquêmico.^26^

As anormalidades lipídicas são uma característica comum nas dislipidemias de diferentes etiologias, sejam genéticas ou secundárias ao uso de medicamentos ou a comorbidades, como diabetes, obesidade e síndrome metabólica.^16 , 17^A hipercolesterolemia que não responde à terapia hipolipemiante convencional, acompanhada de uma leve elevação das enzimas hepáticas, pode levantar suspeita de D-LAL.^27^Na HF, com critérios definitivos ou prováveis, nos quais variantes patogênicas ou provavelmente patogênicas são detectadas, fenocópias, como D-LAL, devem ser descartadas, uma vez que um diagnóstico adequado e um tratamento oportuno podem restaurar ou prevenir alterações hepáticas. Chora et al.^27^ descreveram casos de D-LAL em uma coorte de pacientes com suspeita de HF (n=492), apresentando dislipidemia grave, com diagnóstico genético negativo para genes de HF ou com variantes de significado incerto. As análises de suas famílias também foram realizadas. Nesse estudo, os autores identificaram quatro crianças com D-LAL.^27^

Outra forma de triagem para D-LAL é pela presença de síndrome metabólica com anormalidades hepáticas detectadas por biópsia hepática^28^ ou ultrassonografia sugestiva de MASH ou DHGAM e, raramente, na doença de Wilson sem sinais neurológicos.

Neste estudo, buscamos D-LAL em pacientes adultos (2000 indivíduos) atendidos em um centro de referência para dislipidemias, incluindo pacientes com suspeita de HF (N=92), aqueles com doença hepática (N=168) e em 18 crianças.

O diagnóstico precoce e preciso de D-LAL é crucial para instituir um tratamento adequado e em tempo hábil. Ensaios específicos para D-LAL devem estar disponíveis não apenas para pesquisa, mas também no sistema público de saúde.

Alguns países da América Latina desenvolveram algoritmos para diagnosticar D-LAL,^29 , 30^ e o Brasil incluiu a pesquisa por D-LAL na Atualização das Diretrizes Brasileiras para Dislipidemia e Prevenção da Aterosclerose.^19^ No entanto, a implementação dessa medida ainda é ausente.

O mapeamento dos genes humanos abriu novas perspectivas para o diagnóstico de doenças hereditárias e permitiu o desenvolvimento de estratégias preventivas e terapêuticas.

Ainda há muitas questões a serem respondidas sobre o diagnóstico de D-LAL. A conscientização sobre a doença, o diagnóstico precoce e o tratamento adequado podem impedir a progressão da aterosclerose e da disfunção hepática. Em relatos de casos de indivíduos afetados, a experiência com a terapia de reposição enzimática demonstrou uma melhora considerável nos sintomas e na qualidade de vida, com poucos eventos adversos e um impacto positivo para os pacientes e cuidadores. O maior benefício, porém, foi o aumento da expectativa de vida.^31 , 32^

Até o momento, não há estudos suficientes sobre a prevalência da D-LAL no Brasil, e a doença continua subdiagnosticada. A D-LAL apresenta sinais e sintomas que são comuns a de outras doenças que afetam o fígado, baço e perfil lipídico. A atividade da LAL é variável, e os diferentes fenótipos, doença de Wolman e CESD, possuem apresentações distintas, progressão da doença e mortalidade.

### Pontos fortes e limitações do estudo

Pontos fortes: este estudo foi conduzido em um centro de referência para dislipidemias, utilizou um algoritmo de triagem previamente descrito, e incluiu 2018 pacientes. A D-LAL é uma doença rara, mas apresenta manifestações comuns, como LDL-C elevado, HDL-C baixo, níveis aumentados de AST/ALT e esteatose hepática, características frequentemente observadas em centros de tratamento de dislipidemias.

Limitações: O estudo foi retrospectivo, as enzimas hepáticas foram obtidas sob tratamento com estatinas e a atividade da LAL não foi avaliada em leucócitos. Não testamos a discriminação ou precisão do algoritmo. Na verdade, a D-LAL é muito mais um diagnóstico de exclusão, na investigação de HF ou outras dislipidemias genéticas.

## Conclusão

A triagem de D-LAL em um centro de referência para dislipidemias, utilizando um algoritmo que inclui medidas clínicas e laboratoriais, selecionou 21 pacientes de alto risco. No entanto, a atividade enzimática da LAL não confirmou a doença em oito indivíduos testados. A partir de uma mãe suspeita, uma criança teve a confirmação post-mortem de D-LAL, o que possibilitou o diagnóstico da mãe e de três meio-irmãos afetados.

Identificar a LAL-D continua sendo um desafio, e esse algoritmo pode auxiliar na seleção de pacientes de alto risco para triagem da doença. No entanto, esse algoritmo não foi validado quanto à precisão ou capacidade discriminatória. Dada sua raridade e características sobrepostas com outras dislipidemias genéticas, a LAL-D é, principalmente, um diagnóstico de exclusão, frequentemente considerado quando outras condições tenham sido descartadas.
