# Supplementary material for: Triagem de Deficiência de Lipase Ácida Lisossômica em uma Unidade Clínica de Dislipidemias
Source: Arq Bras Cardiol. 2025 Jul 29;122(7):e20250825. [Article in Portuguese] doi: 10.36660/abc.20240825 (PMC12981353; doi:10.36660/abc.20240825)
Supplement: Translation

# Translation: Screening for Lysosomal Acid Lipase Deficiency in a Lipid Clinic

**DOI:** 10.36660/abc.20240825i

**Authors:** Zenia Brasil, Francisco Antonio H. Fonseca, Marco Antonio Curiati, Sandra Obikawa Kyosen, Vanessa Gonçalves Pereira, Waleria Toledo Fonzar, João Bosco Pesquero, Francy Reis da Silva Patrício, Marcelo Hideki Yamamoto, Joyce Umbelino Yamamoto, Vania D’Almeida, Ana Maria Martins, Maria Cristina Izar

**Affiliations:** 1 Brazil Disciplina de Cardiologia, Departamento de Medicina, Universidade Federal de São Paulo (UNIFESP), São Paulo, SP – Brazil; 2 Brazil Brazilian Network of Collaboration and Knowledge Advancement on Severe Hypertriglyceridemia – Hypertri Brazil Network, Casa dos Raros, Porto Alegre, RS – Brazil; 3 Brazil Centro de Referência em Erros Inatos do Metabolismo (CREIM), Departamento de Pediatria, Universidade Federal de São Paulo (UNIFESP), São Paulo, SP – Brazil; 4 Brazil Laboratório de Erros Inatos do Metabolismo, Universidade Federal de São Paulo (UNIFESP), São Paulo, SP – Brazil; 5 Brazil Departamento de Biofísica, Universidade Federal de São Paulo, São Paulo, SP – Brazil; 6 Brazil Departamento de Patologia, Universidade Federal de São Paulo (UNIFESP), São Paulo, SP – Brazil

**Keywords:** Lipase, Dyslipidemias, Hepatomegaly, Splenomegaly

## Abstract

**Background:**

Lysosomal acid lipase deficiency (LAL-D) is a rare autosomal recessive disease, with massive accumulation of cholesteryl esters and triglycerides in many organs, leading to hepatosplenomegaly, microvesicular steatosis, cirrhosis and premature death. Early recognition is crucial for timely enzyme replacement therapy.

**Objectives:**

To screen for LAL-D in subjects with dyslipidemias and/or liver disease at an outpatient lipid clinic.

**Methods:**

We retrospectively assessed records from 2,018 adults and children using a screening algorithm including ALT/AST elevation >1.5 x upper limit of normality, LDL-C>160 mg/dL, HDL-C<40 (males) or <50 mg/dL (females) in adults, and LDL-C>130 mg/dL, HDL-C<45 mg/dL, in children. High-risk patients for LAL-D were selected for LAL enzymatic activity assay in dried blood spots using LAL inhibitor, Lalistat-2.

**Results:**

Among 2,018 screened patients, 21 (0.92%) were selected for LAL activity test, but only eight performed the test with normal results [mean LAL activity 0.077 ± 0.03 nmol/punch/h (reference value >0.024 nmol/punch/h)]. A child whose mother did not perform the test, had a post-mortem undetectable LAL activity. Further, the mother and three half-brothers confirmed LAL-D. Sequencing (NGS) of LIPA gene did not find pathogenic variants, not allowing to discard changes in non-coding region of the gene analyzed.

**Conclusions:**

Identifying LAL-D remains a challenge, and an algorithm based on clinical and laboratory criteria may assist in selecting patients for LAL-D screening. Given its rarity and overlapping features with other genetic dyslipidemias, LAL-D is primarily a diagnosis of exclusion, often considered when other conditions have been ruled out.

## Introduction

Lysosomal acid lipase (LAL) is the enzyme responsible for intracelular hydrolysis of cholesteryl esters and triglycerides. Deficiency of LAL (LAL-D)^1^is characterized by accumulation of cholesteryl esters and triglycerides, but it also leads to increase in cholesterol synthesis.^2^LAL-D (OMIM # 278000) is a rare autosomal recessive lysosomal storage disease, arising from variants in the *LIPA* gene (OMIM 613497), mapped to chromosome 10q23.2, with 10 exons and approximately 45 kb in length.^3^Affected individuals are typically either homozygous or compound heterozygous for *LIPA* variants.

The disease manifests in two different forms. Wolman disease,^4^in infants, evolves with aggressive symptoms and signs, such as massive accumulation of cholesteryl esters and triglycerides in the liver, spleen, bone marrow, adrenal glands, lymph nodes, intestinal villi, and infiltration of the vascular endothelial, and skeletal muscle. Calcification in the adrenal glands is a common feature of LAL-D and can occur in 50% of infants.^5 , 6^Failure to thrive, due to malabsorption, hepatosplenomegaly, and hepatic failure, due to fibrosis and cirrhosis can be present. Untreated, these complications contribute to the death in the first year of life.^7 , 8^Prevalence studies evaluating this severe phenotype are scarce and vary according to ethnicity and geographic localization from 1:350,000 to 1:528,000 in children, with absent or <1% LAL activity, and usually the individuals are homozygous for pathogenic *LIPA* variants.^9^

Another phenotype of LAL-D is the cholesteryl ester storage disease (CESD)^10^with a varying presentation, depending on LAL activity, ranging from 1-12% of the normal value.^11^This subtype is less recognized, with a slow progression and can be confounded with other disorders with similar clinical and laboratory findings, such as familial hypercholesterolemia (FH), familial combined hyperlipidemia (FCH), metabolic dysfunction-associated steatohepatitis (MASH), metabolic-associated fatty liver disease (MAFLD), or cryptogenic cirrhosis.^12^This phenotype is usually due to double or compound heterozygous pathogenic variants, or attributed to a high polygenic score; affected individuals do not present signs of CESD until childhood or adulthood.^13^Main features of CESD are abdominal distension, esophageal varices, hepatosplenomegaly, coronary artery disease, diarrhea, stroke, malnutrition, and failure to thrive.^5^Bernstein et al.^6^found that hepatic dysfunction and dyslipidemia were the most frequent signs at all ages in CESD, and hepatomegaly and hepatosplenomegaly are the most frequent signs of disease.

Regarding treatment, lipid-lowering therapy with statins seems ineffective for patients with hepatic disorder, as it increases the cholesteryl ester supply to hepatocytes,^13 , 14^thus worsening the disease.^15^However, it is the best option to reduce low-density lipoprotein cholesterol (LDL-C) concentrations, cholesterol synthesis and cardiovascular risk.^16^In addition, in LAL-D there is an impaired regulation of ABCA1 gene, thus leading to low concentrations of HDL-C.^17^Enzyme replacement therapy with sebelipase alpha enables patients to achieve physiological levels of LAL and can prevent cholesteryl ester and triglycerides accumulation.^4 , 18^

Some countries developed guidelines to increase awareness and diagnosis of LAL-D.^19^In our country,^19^LAL-D was included as lysosomal disease and we follow a diagnostic algorithm ( Central Illustration ), previously used as a screening tool.^20^In this article, we describe the screening methods to detect LAL-D in children and adults in a tertiary lipid clinic. We searched for liver disease, hepatic dysfunction plus dyslipidemia, or lipid changes alone. This screening method was based on previous diagnosis of patients with confirmed LAL-D, selected from a postmortem index case with affected family members at the Reference Center for Inborn Errors of Metabolism (CREIM), Universidade Federal de São Paulo.^20^

The aim of this study was to investigate the prevalence of LAL-D in an outpatient population of patients with dyslipidemias, hepatic dysfunction or both based on previous confirmed cases that used this screening method.

## Methods

### Study design

This is a retrospective cross-sectional study, with data obtained from medical records (paper and/or electronic records) of adult and pediatric patients from the outpatient clinics of the Lipids, Atherosclerosis and Vascular Biology Section, Cardiology Division, Universidade Federal de São Paulo. The study protocol was approved by our local ethics committee (research ethics committee of UNIFESP, CAAE: 51989915.2.00005505), and data were collected for 18 months.

We reviewed 2,000 consecutive medical records from adult patients and 18 pediatric patients between August 2017 and February 2019. Among those, there were 92 patients with definite/probable diagnosis of familial hypercholesterolemia (Dutch Lipid Clinic Network), but with negative results for a panel of genes for FH ( *LDLR* , *APOB* , *PCSK9* , *LDLRAP-1* ) plus the *LIPA* gene,^21^and 168 patients with liver disease. Clinical and laboratory data from these patients were obtained to selectively screen for LAL-D.

### Inclusion criteria

- Adult, non-obese (body mass index, BMI<30 Kg/m^2^), with LDL-C >160mg/dL, high-density lipoprotein cholesterol (HDL-C) <40 mg/dL in men and <50 mg/dL in women, and with persistently high alanine aminotransferase (ALT) and/or aspartate aminotransferase (AST) (>1.5 x the upper limit of normality), with or without hepatomegaly.
- Patients with steatosis by ultrasound or mixed or microvesicular steatosis observed on liver biopsy.
- Patients with suspected familial hypercholesterolemia (FH) using the Dutch Lipid Clinic Network criteria^22^without confirmation of FH causing variants on sequencing, or with variants in *LIPA* gene, with ALT and/or AST >1.5x ULN.
- Pediatric patients (<18 years-old) with hepatomegaly on physical examination or imaging tests (with or without splenomegaly), high LDL-C (>130 mg/dL), low HDL-C (<40 mg/dL), or persistently elevated ALT and/or AST (>1.5x upper limit of normality), and/or signs of fibrosis, cirrhosis, or evidence of storage disease on liver biopsy.
- Patients with suspicion of Wilson Disease without neurological involvement.

Exclusion criteria

- Adult patients with steatosis or cirrhosis of known etiology;
- Patients with viral hepatitis and/or alcoholic liver disease;
- Patients with known drug toxicity.

### Database organization

The data collected included patient identification, demography, presence of cardiovascular risk factors, including dyslipidemia, habits, history of cardiovascular disease, liver diseases, metabolic syndrome, and other comorbidities. Clinical and anthropometric parameters were recorded. Laboratory variables included total cholesterol, HDL-C, LDL-C, non-HDL-C, triglycerides (TG), ALT, AST, blood glucose, thyroid-stimulating hormone (TSH), and free thyroxin (T4). In subjects taking statins, LDL-C was corrected by the statin intensity, as described below.^23^Liver and spleen ultrasounds, as well as hepatic biopsies were recorded for abnormalities, when available. Previous laboratory tests or genetic confirmation for LAL-D were also recorded when available. For pediatric patients a separate database was made.

### Treatment with statins

The values of total- and LDL-C of adult patients were corrected by the intensity of statin treatment, multiplying the total- and LDL-C value by a factor: high-intensity, LDL-C reduction >50% (x 2.00); moderate-intensity, LDL-C reduction between 30-50% (x 1.65), and low-intensity, LDL-C reduction ~ 30% (x1.43).^23^High intensity treatment was defined as atorvastatin 40/80 mg, rosuvastatin 20/40mg, simvastatin 20/40 mg plus ezetimibe; moderate intensity, atorvastatin 10/20 mg, rosuvastatin 5/10 mg, simvastatin 10/40 mg; low-intensity as other statins or lower doses.

### Biochemical diagnosis

Subjects who met the inclusion/exclusion criteria were invited to collect blood samples for determination of LAL activity ( Figure 1 ). These patients signed the written informed consent form prior to blood collection.

**Figure 1:** – Profile of patients with dyslipidemia from medical records according to intensity of statin treatment (a) and those with inclusion criteria for LAL-D (b). High intensity: atorvastatin 40/80 mg, rosuvastatin 20/40mg, simvastatin 20/40 mg + ezetimibe 10 mg; moderate intensity: atorvastatin 10/20 mg, rosuvastatin 5/10 mg, simvastatin 10/40 mg; low-intensity: other statins or lower doses.

LAL enzymatic activity assay was obtained from dried blood spot (DBS) samples according to the fluorimetric technique described by Hamilton et al.^24^and performed at *Laboratório de Erros Inatos do Metabolismo* (LEIM) – UNIFESP, which used the reference range of >0.024 nmol/punch/h. The assay was performed in duplicates, with positive and negative controls.

### Genetic study for detection of familial hypercholesterolemia

DNA extraction, next-generation sequencing and variant detection analyses were performed according to Fonzar et al.^21^

### Data analysis

Descriptive statistics were used in this research. Categorical variables were described as frequencies and percentages; numerical data were presented as means ± standard deviation (SD) or median and interquartile range (IQR) as appropriate. Normality was tested by Kolmogorov-Smirnov test. Significance was established at a P-value < 0.05. All statistical analyses were performed using the Statistical Package for the Social Sciences (SPSS, Chicago, IL) version 22.

## Results

### Descriptive analysis of the adult population

Characteristics of the adult population are presented in Table 1 and included the FH adult cohort. Of the 2,000 patient records evaluated, 893 individuals (44.6%) were receiving high-intensity treatment, 529 (26.4%), moderate intensity, four (0.2%), low-intensity, and 574 (28.8%) were not taking statins. Of those, 21 patients had the inclusion criteria for LAL activity screening ( Figure 1 ), 14 in high-intensity treatment, four in moderate-intensity, and three not taking statins.

**Table 1:** – Clinical and laboratory characteristics of the adult population

Characteristic	N=2,000
Age, years	67 (60-74)
Sex Male/Female, %	613 (30.65) / 1387 (69.35)
Race, %	
Caucasian	1022 (51.10)
Black	197 (9.85)
Mixed	781 (39.05)
Hypertension, %	1528 (76.4)
Dyslipidemia, %	1615 (80.75)
Diabetes, %	778 (38.9)
Metabolic syndrome, %	595 (29.75)
Statin intensity, %	
High-intensity	893 (44.6)
Moderate-intensity	529 (26.4)
Low-intensity	4 (0.2)
No statin	574 (28.8)
Myocardial infarction	215 (10.75)
Smoking, %	
Current	110 (5.50)
Former	467 (23.25)
BMI, kg/m^2^	30.88 (24.65-40.00)
Total cholesterol, mg/dL	227 (152-279)
HDL-C, mg/dL	49 (40-59)
LDL-C, mg/dL	156 (115-204)
Non-HDL-c, mg/dL	121 (96-155)
Triglycerides, mg/dL	124 (99-177)
AST, U/L	48 (48-60)
ALT, U/L	49 (49-61)
Glucose, mg/dL	102 (94-118)
HbA1c, %	6.10 (5.70-6.70)
TSH, mUI/L	2.65 (1.76-4.21)
T4, ng/dL	1.30 (1.14-1.51)

Categorical variables are expressed as frequencies (%); numerical variables expressed as median (IQR); correction for intensity of treatment was made multiplying total cholesterol or LDL-C by 1.43 (low-intensity), 1.65 (moderate intensity) and 2.00 (high intensity). HDL-c: high-density lipoprotein cholesterol; LDL-C: low-density lipoprotein cholesterol; ALT: alanine aminotransferase; AST: aspartate aminotransferase; IQR: interquartile range; BMI: body mass index; TSH: thyroid-stimulating hormone; T4: free thyroxin.

The cohort of 92 patients with definite or probable diagnosis of FH (Dutch Lipid Clinic Network) was also selected.^22^Among those, we found two patients with variants of uncertain significance (VUS) on *LIPA* gene, normal liver enzymes and no signs of hepatic steatosis. These patients were not screened for LAL activity.

### Descriptive analysis of pediatric patients with criteria for LAL-D

Eighteen patients were retrieved from the medical records of our lipid outpatient clinic and the CREIM ( Table 2 ). Of those, eight (44%) had LDL-C above target, and three (17%) had HDL-C <40 mg/dL; in nine patients, AST and/or ALT were high. All criteria were present in five (28%) patients. One of these patients had genetic confirmation for Niemann-Pick disease, another had an inherited mitochondrial disease and were not selected for LAL-D screening.

**Table 2:** – Characteristics of children screened for lysosomal acid lipase deficiency

Variable	Total (N=18)
Age, years	12 (7-18)
Male/female, %	12 (67)/6 (33)
Race, %	
Caucasian	10 (55)
Black	1 (6)
Mixed	7 (39)
Lipid-lowering therapy, %	
High-intensity	0 (0)
Moderate-intensity	1 (5)
Low-intensity / no therapy	1 (5)
No statins	16 (90)
Body weight, Kg	38 (26-48)
BMI, Kg/m^2^	18 (17-19)
Heart rate, bpm	70 (67-76)
Total cholesterol, mg/dL	213 (135-329)
HDL-C, mg/dL	44 (21-64)
LDL-C, mg/dL	152 (67-250)
Non-HDL-C, mg/dL	170 (82-265)
Triglycerides, mg/dL	91 (52-167)
Fasting glucose, mg/dL	88 (77-103)
HbA1c, %	5.5 (4.8-6.3)
AST (U/L)	27 (18-66)
ALT (U/L)	19 (10-62)
CK (U/L)	135 (110-241)
TSH (µIU/L)	3.30 (1.24-6.12)

Categorical variables are expressed as frequencies (%); numerical variables expressed as median (IQR). ALT: alanine aminotransferase; AST: aspartate aminotransferase; BMI: body mass index; HDL: high-density lipoprotein; IQR: interquartile range; LDL: low-density lipoprotein; SD: standard deviation; CK: creatine phosphokinase; TSH: thyroid-stimulating hormone.

### Descriptive analysis of patients with hepatic steatosis

Table 3 presents the characteristics of 168 patients with hepatic steatosis, either by ultrasound or hepatic biopsy. One-hundred and fifty-five (92.26%) patients presented steatosis at ultrasound, parenchymal hepatopathy was found in 12 (7.14%), and hepatic nodule in 1 (0.59%). From these findings we selected patients with corrected LDL-C >160 mg/dL and HDL-C <40 mg/dL in males or <50 mg/dL in females, AST or ALT >1.5x the ULN or hepatic steatosis by imaging methods.

**Table 3:** – Distribution of ultrasound findings and/or hepatic biopsy in the participants of the study

Variable	N=168
Steatosis (%)	155 (92.26)
Parenchimal hepatopathy (%)	12 (7.14)
Hepatic nodule (%)	1 (0.59)

Categorical variables are expressed as frequencies (%).

Analysis of patients with Wilson Disease without neurological involvement was not performed because there were no subjects with this criterion.

### LAL-activity

From the selected databases, 21 (0.92%) patients had the inclusion criteria for LAL-D screening with LAL activity (three children and 18 adults); mean ALT/AST were, respectively, 83.7 and 96.7 U/L, mean total cholesterol was 248 mg/dL, LDL-C 222 mg/dL, and four had documented hepatic steatosis. Of those, two subjects did not agree to participate, three died, and it was not possible to contact eight patients, including the children. Eight patients signed the written informed consent and collected blood for the assay. Six were women, and two men aging 58-82 years; in 100% ALT was above 1.5 x upper limit of normality, two had hepatic steatosis, and all were taking high-intensity statins. No significant differences were found for patients selected and screened for LAL-D (Supplemental Table S1).

The assay for LAL activity was performed in duplicates plus two positive and two negative controls. Mean LAL activity was normal (0.077 [± 0.03] nmol/punch/h, reference value >0.024 nmol/punch/h). Negative controls presented 0.096 nmol/punch/h and positive controls showed undetectable LAL activity ( Table 4 ).

**Table 4:** – Results of lysosomal acid lipase (LAL) activity in enzimatic assay with the Lalistat-2 inhibitor

Sample	Well	Values	Mean	SD	V%	Without - with	pmol/punch/L	Straight Line 4/11
01	C2 D2	2698.469 2714.956	2706.713	11.658	0.4	332.211	40.991	45.558
02	C3 D3	2794.832 2643.668	2719.250	106.889	3.9	488.673	62.988	73.908
03	C4 D4	2835.840 2881.884	2858.862	32.558	1.1	702.193	92.940	112.511
04	C5 D5	2867.881 2931.860	2899.871	45.240	1.6	651.496	85.885	103.418
05	C6 D6	2707.930 2725.490	2716.710	12.417	0.5	546.769	71.117	84.385
06	C7 D7	2781.802 2832.713	2807.258	36.000	1.3	602.909	78.958	94.490
07	C8 D8	2782.242 2791.745	2786.994	6.720	0.2	608.951	79.848	95.638
08	C9 D9	2824.358 2847.077	2835.718	16.065	0.6	776.723	103.405	125.998
+ Control	G10 H10	2693.484 2824.164	2758.824	92.405	3.3	-40.715	-11.371	-21.928
- Control	G9 H9	2806.659 2734.641	2770.650	50.924	1.8	729.464	96.769	117.446

Tests were performed in duplicates for each patient and a positive (+ Control) and negative controls (- Control); LAL-activity performed with the inhibitor lalistat-2; Results shown in nmol/punch/h (reference value >0.024 nmol/punch/h).

We tried to find more details of these patients, and a 41-year-old woman, whose LAL activity was previously normal, repeated the test for LAL activity after one of her children had died. LAL activity was 0.00 nmol/punch/h in two further tests. She presented hepatomegaly, hepatic steatosis, confirming LAL-D. Her nine-year-old daughter had hepatosplenomegaly and hepatic steatosis, AST and ALT levels of 55 and 35 U/L, respectively, total cholesterol and LDL-C of 212 mg/dL and 148 mg/dL, respectively; HDL-C 12 mg/dL, non-HDL-C 200 mg/dL, and TG 256 mg/dL. The onset of symptoms occurred at three years old; she presented with intermittent diarrhea, and failure to thrive. At the age of four, her body weight was 16 Kg (z score of −0.56), and her height was 93 cm (z score of −3.0). Liver biopsy showed grade 3 macro- and micro- vesicular diffuse steatosis in 60% of the hepatocytes, enlargement of portal spaces with enlarged micro vacuolized macrophages and hepatocytes. By age nine, she developed progressive hepatosplenomegaly and worsening of diarrheic episodes. She underwent several hospitalizations due to respiratory distress secondary to severe hepatosplenomegaly and died at age nine due to pneumonia and septic shock.

Differential diagnosis of LAL-D for the deceased child included analyzing blood samples and liver biopsies. Three liver biopsies at different hospitals showed macro- and micro-vesicular steatosis, with a pattern similar to that of Niemann-Pick C disease.

Blood samples were collected but were analyzed post-mortem. The tests were designed to assess enzyme activity associated with various lysosomal storage disorders. Chitotriosidase activity was measured in plasma, using an enzyme-linked immunosorbent assay (ELISA); sphingomyelinase activity was assessed in leukocytes to rule out Niemann-Pick disease; beta-glucosidase activity was measured in leukocytes to evaluate for Gaucher disease; a comprehensive panel obtained by dried blood spots tested for chitotriosidase, sphingomyelinase, beta-glucosidase, and lysosomal acid lipase activity, providing a broader assessment for lysosomal storage diseases. All the tests, except for LAL-D were within the normal range. Undetectable enzymatic activity (0 nmol/punch/h) was observed (reference range >0.024 nmol/punch/h), confirming the diagnosis of LAL deficiency. These enzyme assays were essential in differentiating LAL-D from other lysosomal storage disorders with overlapping clinical features.

Her autopsy revealed disseminated lipid and cholesterol crystal accumulation in the liver, spleen, bone marrow, and gastrointestinal tract, and severe steatosis with lipid-filled histiocytes. No adrenal calcification was observed. This girl was the index case of her family, second of five siblings from the same mother, who has a son (unaffected) and a daughter (this patient) from her first marriage, and three other sons (all affected), from her second marriage. Both marriages were nonconsanguineous unions, and both fathers are not related.

Screening of the three male half-brothers was performed, and they presented LAL activity of 0.0087 nmol/punch/h, 0.00 nmol/punch/h, and 0.00 nmol/punch/h, confirming the diagnosis of LAL-D. Their case reports have been previously published.^20^We further performed genetic tests of the mother and three half-brothers of the index case obtaining DNA extracted from saliva. Genetic sequencing (NGS) using a panel that included the *LIPA* gene did not find any pathogenic variants, not allowing to discard changes in the non-coding region of the genes analyzed, such as regulatory regions, intergenic and intronic sequences distant from the exons.

## Discussion

In this study we investigated the prevalence of LAL-D in an outpatient population of subjects with dyslipidemias, and used an algorithm based on previously confirmed clinical cases that were diagnosed using the enzymatic assay for LAL activity.

The disease is reported to be very rare, with cholesteryl ester accumulation in tissues like liver, spleen, bone marrow, adrenal glands, lymph nodes, intestinal villi, vascular wall, and skeletal muscles.^5 , 6^A more severe form is recognized in children, and less pronounced manifestations can be seen in adults, depending on LAL enzymatic activity.^4^In our study, in the lipid clinic, in 2,018 individuals we found 21 (0.92%) at high-risk for LAL-D, and among the eight that were initially tested for LAL activity, we could not confirm any case. However, a post-mortem positive test in a child (index case) confirmed the mother and three affected half-brothers.

The Update of Brazilian Guidelines for Dyslipidemia and Atherosclerosis Prevention^19^recognize the gap in the diagnosis of the disease and suggests an algorithm to screen for LAL-D ( Central Illustration ). Individuals with LAL-D experience great burden of disease, and according to their reports, symptoms are constant, including abdominal pain, hepatosplenomegaly, headache, weakness, pruritus, skin lesions.^20^In children, the phenotype can be present in the first months of life, with abdominal enlargement, failure to thrive, hepatomegaly or hepatosplenomegaly, hepatic steatosis, and adrenal calcification.^20 , 25^

There is a huge spectrum of manifestations of the disease of late onset. Symptoms can be mild and not be perceived, leading to underdiagnosis. On the other hand, in the most severe cases, liver alterations can lead to hepatic cirrhosis and death.^5^A higher cardiovascular risk, with history of atherosclerotic cardiovascular disease or ischemic stroke can be present.^26^

Lipid abnormalities are a common feature in dyslipidemias of different etiologies, either genetic or secondary to certain drugs or comorbidities, such as diabetes, obesity, metabolic syndrome.^16 , 17^Hypercholesterolemia that does not respond to conventional lipid-lowering therapy, with mild elevation of liver function enzymes, can raise suspicion of LAL-D.^27^In FH, with definite or probable criteria, in whom pathogenic or probably pathogenic variants are detected, phenocopies, such as LAL-D must be ruled out, because appropriate diagnosis and timely treatment can restore or prevent liver alterations. Chora et al.^27^described cases of LAL-D in a cohort of patients with suspected FH (n=492), presenting severe dyslipidemia, with negative genetic diagnosis for FH genes, or with VUS. The analyses of their families were also performed. In that study the authors identified four children with LAL-D.^27^

Another way to screen LAL-D is by the presence of metabolic syndrome with liver abnormalities in hepatic biopsy^28^or ultrasound suggestive of MASH or MAFLD and, rarely, in Wilson’s disease without neurological signs.

In this study we searched for LAL-D in adult patients (2,000 subjects) assisted in a reference center for dyslipidemias, including patients with suspected FH (N=92), those with hepatic disease (N=168), and in 18 children.

Early and accurate diagnosis of LAL-D is crucial to institute appropriate and timely treatment. Specific assays for LAL-D should be available not only for research, but also in the public health system.

Some countries in Latin America developed algorithms to diagnose LAL-D,^29 , 30^and Brazil has included the search for LAL-D in the Update of Brazilian Guidelines for Dyslipidemia and Atherosclerosis Prevention.^19^However, implementation of this measure is lacking.

### Mapping human genes opened new perspectives for the diagnosis of inherited diseases and allowed preventive and therapeutic strategies.

There remain many questions to be answered regarding diagnosis of LAL-D. Disease awareness, early diagnosis and treatment can refrain atherosclerosis and hepatic dysfunction progression. In case reports of affected individuals, the experience with enzyme replacement therapy has shown considerable improvement in symptoms and quality of life, with few adverse events and a positive effect for patients and care givers. The greatest achievement was the increase in life expectancy.^31 , 32^

To date there are not sufficient studies on prevalence of LAL-D in Brazil, and the disease remains underdiagnosed. LAL-D has signs and symptoms that are seen in other diseases that affect the liver, spleen, and the lipid profile. LAL activity is variable and the different phenotypes, Wolman disease and CESD, have distinct presentation, disease progression and mortality.

### Study strengths and limitations

Strenghts: this study was performed in a reference center for dyslipidemias, used a previously described screening algorithm, and included 2,018 patients. LAL-D is a rare disease, but presents common manifestations, such as high LDL-C, low HDL-C, increased leves of AST/ALT, and hepatic steatosis, which are common features in a lipid clinic.

Limitations: the study was retrospective, liver enzymes were obtained under statin treatment, and LAL activity was not performed in leukocytes. We did not test discrimination or accuracy of the algorithm. In fact, LAL-D is much more an exclusionary diagnosis, when testing for FH or other genetic dyslipidemias.

## Conclusion

Screening for LAL-D in a reference center for dyslipidemias using an algorithm including clinical and laboratory measures, selected 21 high-risk patients. However, enzymatic LAL activity did not confirm the disease in eight tested subjects. From a suspected mother, a child had a post-mortem confirmation of LAL-D, that further enabled the diagnosis of the mother and three affected half-brothers.

Identifying LAL-D remains a challenge, and this algorithm may assist in selecting high-risk patients for LAL-D screening. Nevertheless, this algorithm has not been validated for accuracy or discriminatory ability. Given its rarity and overlapping features with other genetic dyslipidemias, LAL-D is primarily a diagnosis of exclusion, often considered when other conditions have been ruled out.
